# Participatory capacities and vulnerabilities assessment: Towards the realisation of community-based early warning system for deep-seated landslides

**DOI:** 10.4102/jamba.v11i1.555

**Published:** 2019-04-16

**Authors:** Brian A.L. Gumiran, Fatima M. Moncada, Harianne J. Gasmen, Nathalie R. Boyles-Panting, Renato U. Solidum

**Affiliations:** 1 Department of Science and Technology, Philippine Institute of Volcanology and Seismology, Quezon City, Philippines; 2 Disaster Risk Reduction and Climate Change, Department of Science and Technology, Philippine Institute of Volcanology and Seismology, Quezon City, Philippines

## Abstract

Existing frameworks of community-based early warning systems (CBEWS) lack focus on its actual implementation. Furthermore, they do not describe how a top-down early warning system (EWS) can be transformed into a CBEWS. Hence, to support the establishment of a community-based early warning system for deep-seated landslides (CBEWS-L), this study aimed to assess the capacities and vulnerabilities of five *barangays* in the Philippines. The CBEWS-L to be established is envisioned to be information and communication technology (ICT)-based. An ICT4D perspective was therefore taken in conducting this participatory study. Stakeholder mapping, focus group discussions and key informant interviews were used to gather data. Validation was also done through triangulation and post-analysis consultations. The results showed that there are varying sets of capacities and vulnerabilities existing in each community. Moreover, ICT capacities are lacking and are outnumbered by vulnerabilities. Yet, site-specific action points to enhance capacities and resolve vulnerabilities were determined. Still, overall strategies were not uncovered. Furthermore, compounding the capacities and vulnerabilities in each site are indirect factors which, if examined thoroughly, may lead to more complex socio-political issues. It is therefore recommended that in establishing a CBEWS, a comprehensive community risk assessment is first conducted to exhaust all possible action points that can be used in formulating site-specific strategies. Moreover, development of technological solutions must be modular to allow flexibility in accommodating complex community capacities and vulnerabilities.

**Keywords:** Early warning systems; community risk assessment; participatory research; information and communication technology or development; community-based disaster risk reduction and management.

## Introduction

Deep-seated landslides are characterised by a slip surface of at least 3 m (Kaunda 2010). Their occurrences are typically related to about 30–90 days of constant rainfall, as opposed to shallow landslides which are often related to 1–15 days of rainfall (Zêzere, Trigo & Trigo 2005). This makes the Philippines, a country frequently visited by typhoons, prone to deep-seated landslides.

Compared to shallow ones, deep-seated landslides are said to pose larger risks, as they are more likely to cause disastrous debris flows and landslide dams (Dou et al. 2015). Such was the case of the 2006 Southern Leyte Landslide that recorded 139 casualties (Catane et al. 2007).

Aside from larger risks, deep-seated landslides are also characterised by slow movements and varying triggers (Kaunda 2010). Garcia and Fearnley (2012) highlighted the need for continuous monitoring of such long-return-period hazards. Hence, the need for sustainable monitoring techniques for deep-seated landslides is apparent (Garcia & Fearnley 2012; Kaunda 2010). Furthermore, for hazard information to be actionable, it should be relayed to the community as warnings to prevent imminent danger (International Federation of Red Cross and Red Crescent Societies 2012).

In the Philippines, a monitoring system for deep-seated landslides was initiated in 2008 (Marciano et al. 2011). The system uses tilt and moisture sensors to measure parameters and transmit data over global system for mobile communications (GSM) cellular infrastructure. To further involve the community, the monitoring system was expanded to an early warning system (EWS) (Marciano et al. 2014). There were 50 at-risk *barangays* (villages) which were provided with it. The EWS integrated the monitoring system with community-based monitoring of surficial measurements conducted by the local landslide monitoring committee (LLMC). Local landslide monitoring committee is a volunteer group initially organised by the system implementers. Monitored and analysed data became the basis of early warning information (EWI) sent to the community and the local government units (LGUs).

However, the EWS is centrally operated by a national government institution – Department of Science and Technology – Philippine Institute of Volcanology and Seismology (DOST-PHIVOLCS). Department of Science and Technology – Philippine Institute of Volcanology and Seismology makes use of information and communication technology (ICT) such as computers to collect data from the sites, analyse the risk and generate warning. Early warning information is then sent via short message service (SMS). However, as most communities catered by the EWS are located in far-flung areas, this manner of data transfer and information dissemination results in delay in warning issuance. Hence, there is a need to properly conceptualise the EWS in a manner that appropriately involves the community.

Although there is no single definition of the term (Macherera & Chimbari 2016), EWS is commonly defined as:

… the set of capacities needed to generate and disseminate timely and meaningful warning information to enable individuals, communities and organisations threatened by a hazard to prepare and to act appropriately and in sufficient time to reduce the possibility of harm or loss. (United Nations International Strategy for Disaster Reduction 2009)

United Nations International Strategy for Disaster Reduction (UNISDR) (2009) also conceptualised a people-centred approach to EWS. Using said approach, participation of locals is advocated across four key EWS elements: risk knowledge, monitoring and warning of hazards, communication and dissemination of alerts and the local response capabilities.

The people-centred EWS (PEWS) is related with the community-based early warning systems (CBEWS) concept. The latter is defined as the capability of a community to systematically collect, compile and/or analyse information that is used to provide recommended actions to reduce harm or loss from a hazard event (IFRC 2012).

Community-based early warning systems can be more cost-effective and politically viable than other strategies such as relocation (Pellini et al. 2013). It can be implemented through the integration of indigenous practices (Fischer et al. 2012) and employment of simple methodologies (Abon, David & Tabios 2012). Moreover, it can be a sustainable solution through involving the local communities in shaping and implementing the system. This enables operation even years after government interventions in the site (Garcia & Fearnley 2012; Macherera & Chimbari 2016).

Macherera and Chimbari (2016) observed that an EWS is more sustainable when it is owned and operated by local communities. This is empowering because it enables threatened communities themselves to prepare for and respond to hazards (Fathani & Karnawati 2012; Fischer et al. 2012; Macherera & Chimbari 2016). Specifically for landslides, Fathani, Karnawati and Wilopo (2016) indicate that as at-risk areas are often isolated, community-based implementations are expected to increase capacities and resilience of the local communities as first responders.

Hence, CBEWS differs from national top-down EWS because it maintains that local communities must be treated as equal participants across all the four elements, where their preferences and needs drive the development and implementation of an EWS (IFRC 2012). A brief analysis of five CBEWS in the literature *vis-à-vis* the four elements of the PEWS is presented in Table 1.

**TABLE 1:** Summary of local participation in five operational community-based early warning systems.

Author	Risk knowledge	Monitoring, warning and service	Dissemination and communication	Response capability
Fischer et al. (2012)	Risk analysis	Gauge reading and monitoring	Operation of bells, *rondas* (house-to-house)	Local government led-responses; evacuation drill
Stone et al. (2014)	Not described	Physical ground observation	Maintenance and management of sirens	Facilitate evacuation
Abon et al. (2012)	Not described	Reading of rain gauge data	Not described	Not described
Manalo (2013)	Ground survey	Reading and analysis of rain gauge data	Ringing of bells	Not described
Gonzales et al. (2013); Pineda (2015)	Not described	Affiliated crowdsourcing (physical observations); local decision-making on warning	Receipt and request of information	Local decision-making on response actions
Fathani and Karnawati (2012); Karnawati et al. (2011)	Village-scale hazard and risk mapping	Operation and maintenance of technical system	Operation and maintenance of technical system (i.e. setting of alarm)	Evacuation drill, formulation of action plan, continuing public education

Note: Please see the full reference list of the article, Gumiran, B.A.L., Moncada, F.M., Gasmen, H.J., Boyles-Panting, N.R. & Solidum, R.U., 2019, ‘Participatory capacities and vulnerabilities assessment: Towards the realisation of community-based early warning system for deep-seated landslides’, *Jàmbá: Journal of Disaster Risk Studies* 11(1), a555. https://doi.org/10.4102/jamba.v11i1.555, for more information.

It is evident from Table 1 that CBEWS implementation varies. This is consistent with claims in the literature that there is no single realising framework for EWS (Macherera & Chimbari 2016; Zia & Wagner 2015).

Another notable observation was that there is little evidence of community involvement in two particular PEWS elements: risk knowledge and response capability. Of these two elements, risk knowledge is more of a prerequisite to establishing a CBEWS. In fact, Fischer et al. (2012) list the conduct of participatory disaster risk assessment (PDRA) as an early step towards the establishment of local flood EWSs in the Philippines. Hence, without clear evidence that locals are involved in the assessment of their own risks, it is then vague whether local preferences and needs were at all considered in the reviewed systems.

Likewise, the EWS-L (EWS for deep-seated landslides) in the Philippines (Marciano et al. 2014) provides limited community involvement (i.e. only in providing data [monitoring] and receiving alerts [communication]). Despite the initial involvement of the community in the EWS-L, there is a necessity to proceed with further localisation through transforming the national EWS into a community-based EWS for deep-seated landslides (CBEWS-L). This transformation is expected not to be a linear handover of ICT to the community, as this may disempower them (Beardon 2008). Therefore, the process requires understanding local contexts to help identify appropriate technologies (Garcia & Fearnley 2012) for the community.

We want to address the gap of the transformation of a top-down EWS for deep-seated landslides into a CBEWS-L. We did this by systematically assessing the local context where the CBEWS-L will be established. Although there are many studies that explained the difference between top-down (national) and bottom-up (community-based) approaches (Fathani & Karnawati 2012; Macherera & Chimbari 2016; Manalo 2013; Pineda 2015; Zia & Wagner 2015), there are limited recommendations about establishing an EWS with the community. There are also limitations in literature on establishing CBEWS for deep-seated landslides because other studies in Southeast Asia focused on shallow landslides (Fathani & Karnawati 2012; Neussner 2015). Moreover, studies highlighting the integration of ICT in the establishment of EWS are lacking. Our study therefore aimed to assess the capacities and vulnerabilities of five deep-seated landslide-prone areas in the Philippines in relation to the establishment of their CBEWS-L.

## Research methods and design

### Research design

The study was a qualitative research on the operation and ownership of a CBEWS-L in Philippine *barangays* at-risk of deep-seated landslides. A participatory methodology that enables local people to analyse their own knowledge of their conditions (Bergold & Thomas 2012; Mercer et al. 2008) was used. The study recognised that people within the social condition of interest are knowledgeable of such conditions and are able to make their knowledge known (Mercer et al. 2008). Being a constructivist research (Mercer et al. 2008), the research also treated the participants as learners who generate their own understanding through guided discovery (Bruner 1961). Researchers and research instruments therefore served only as scaffolds or facilitators (Vygotsky & Cole 1978).

A reframed ICT for Development (ICT4D) Theory also guided the research. ICT for Development holds that for ICT to be empowering, it should be created through participation, ensuring that all voices are heard and that it leads to the realisation of rights (Beardon 2008). Hence, although the current EWS-L consists mostly of ICT, the researchers and participants remained open to non-ICT solutions for the realisation of a CBEWS-L.

Capacities and vulnerabilities assessment (CVA) was the method used. Capacities and vulnerabilities assessment holds that local communities should undergo ‘self-discovery and self-analysis’ so that the study results could effectively inform the CBEWS planning and decision-making (Davis, Haghebaert & Peppiatt 2004). In particular, the study has adapted the framework of CVA that divides capacities and vulnerabilities into three interrelated areas: physical/material, social/organisational and motivational/attitudinal (Anderson and Woodrow in Davis et al. 2004). However, in our study, we integrated the use of ICT as a strategy in assessing the capacity of the community to have their own CBEWS-L. We did this through adding another dimension – human capital. It composed of knowledge and skills that the community uses to achieve their goals including knowledge and skills to use ICT tools to their advantage. In addition, action points to address vulnerabilities and increase capacities were identified.

### Research participants

Capacities and vulnerabilities assessment also adheres to the idea that vulnerabilities and capacities are site-specific (Davis et al. 2004). Community was therefore the unit of analysis. We adapted Allen’s (2006) definition of community: ‘the population living within the territorial bounds of a town or village administrative unit, which is considered to be exposed to a relatively high degree of environmental hazard risk’. In the Philippines, this pertains to the *barangay*, the lowest tier of local government.

The study was conducted in five purposively sampled barangays out of the 50 which the EWS-L currently caters. The study sites were chosen purposively based on: (1) community diversity (indicated by presence of indigenous groups); (2) site accessibility (indicated by presence of roads, highways, cellular and/or radio signals); (3) local government unit (LGU) structures/relations (indicated by perceived structure and relations among residents and LGUs); and (4) community resources (indicated by municipality class). Below is a summary of each sample site’s description:

- Site A – easily accessible through the national highway and has some GSM signal; has reported challenge with LGU structure and relations but belongs to a first-class municipality.
- Site B – composed of an indigenous group; far from the provincial centre and has limited to no GSM signal; also reported to have negative LGU relations and belongs to a second-class municipality.
- Site C – composed of an indigenous group; easily accessible through the national highway and is near the municipality centre; has some GSM signal; has good LGU relations and belongs to a fifth-class municipality.
- Site D1 – far from the municipality centre but has some GSM signal; has good LGU relations and belongs to a second-class municipality.
- Site D2 – part of the same municipality as D1 and is also far from the municipality centre; has limited to no GSM signal.

In each site, the groups that were invited were the barangay council, members of the households at risk (based on the initial hazard mapping) and the LLMC. However, we also included higher tiers of local government disaster risk reduction and management (DRRM) offices (municipal and provincial levels) as supporting stakeholders. The number of participants per stakeholder group was based on their availability, but an effort to include people from vulnerable sectors of each community such as youth and older persons was made. The number of participants per study site was as follows: site A – 36; site B – 28; site C – 25; site D1 – 25; and site D2 – 26.

### Data gathering procedure

To implement the CVA, two workshop activities were used. Firstly, a CVA template adapted from Anderson and Woodrow in Davis et al. (2004) was completed by the participants. An additional category called Human Resources was added to encompass knowledge and skills related to operating a CBEWS-L. Stakeholder influence mapping (Mayers & Vermeulen 2005) was also done to uncover capacities and vulnerabilities of the community in terms of power. This activity focused on three dimensions: influence over decision-making, information access and credibility. Focus group discussions were conducted after each workshop. In addition, representatives of each LGU were selected as key informants for semi-structured interviews to complement the workshops. Field notes, workshop results and interview notes were the primary sources of data.

### Data analysis

*In situ* analyses were facilitated using the integrated CVA template explained in the previous section. Gathered data were further content analysed and categorised. The pressure and release (PAR) model (Wisner et al. 2003) was used as the coding tree. It is used to illustrate how risks are caused by the presence of unsafe conditions that are brought by dynamic pressures and root causes that include several social and political issues. Consequently, addressing dynamic pressures and root causes entails the resolution of these social and political issues.

However, emergent themes were later assigned new codes. The interrelations among codes are described in the lens of the PAR model through the progression of vulnerability through dynamic pressures and root causes, as verified by causal statements in the data. Common themes and relations among the five case studies were also highlighted during the analysis. Finally, a validation fieldwork was performed with the same stakeholders, months after the workshop to confirm the analysis made by the study.

## Ethical considerations

All participants were informed of the study’s objectives, limitations and implications and were recruited on a voluntary basis. Informed consent was secured via phone calls. Furthermore, the methods to be used were described in a series of meetings before the actual data gathering workshop. Confidentiality and anonymity were also maintained.

## Results

With the aim to assess the viability of establishing the CBEWS-L, the capacities and vulnerabilities of five deep-seated landslide-prone areas in the Philippines were identified. The capacities and vulnerabilities presented in tables are grouped according to the following categorisation:

- social relations – existence of a CBEWS-L organisation or coalition who will lead the operations, including their motivational, organisational and other social capacities (i.e. credibility, influence, information access), community motivation and relations
- physical resources – all needed ICTs, public utilities, facilities, EWS-L tools and equipment, and financial resources
- trainings and learning resources – resources educating the community on various EWS processes, including landslide sensor data analysis
- appropriate skills – individual literacy, numeracy, ICT, management and EWS-L technical skills of the community
- governance and accountability – active involvement of LGUs, coordination across LGU levels and willingness of LGUs to help the community.

These categories were lifted from the PAR model, except for the governance and accountability category. Governance and accountability was appropriately coded because the capacities and vulnerabilities falling under this category cannot be related with any of the categories found in the PAR model.

Aside from the tabulated data, the participants were able to formulate action points for their capacities and vulnerabilities. In formulating said action points, further capacities and vulnerabilities were revealed as detailed in the following subsections.

### Summary of results in study site A

While the LLMC leader of site A actively participates in the current EWS-L, members are not as participative. According to the LLMC members, compensation for the LLMC can help resolve the issue. For them, local economy is one hindrance to their participation. Specifically, they would rather spend their hours on their livelihoods than volunteer for LLMC (Table 2).

**TABLE 2:** Categorised capacities and vulnerabilities of site A.

Variable	Social relations	Physical resources	Trainings and learning resources	Appropriate skills	Governance and accountability
Capacities	Active LLMC leader	Available evacuation centre	Protocol trainings from PHIVOLCS	Surficial data gathering	Positive relations between local legislative and executive branches of the MLGU
	-	-	-	High basic literacy and numeracy	-
	-	-	-	Has some computer skills	-
Vulnerabilities	Low credibility of LLMC	Lack of office space and equipment	Lack of training on sensor maintenance	Low management skills	Uncertainty about political will in case of change in elected officials
	Low participation of LLMC members	Limited radio, GSM and Internet connectivity	-	-	Lack of concrete DRRM policies at the barangay level
	No defined structure for information access	-	-	-	-
	Lack of sense of ownership over the EWS	-	-	-	-

DRRM, disaster risk reduction and management; EWS, early warning system; GSM, global system for mobile communications; LLMC, local landslide monitoring committee; MLGU, municipal local government unit; PHIVOLCS, Philippine Institute of Volcanology and Seismology.

Another action point that the participants think will help improve LLMC performance is to increase community awareness of the landslide risk. According to them, only a few are willing to do volunteer work because most residents are unaware of the deep-seated landslide hazard in their community. They do not fully comprehend the risk they are facing. This was also linked to the low credibility of LLMC and lack of sense of ownership over the EWS-L.

On the contrary, the participants attributed their vulnerabilities in terms of physical resources and training and learning resources to lack of government funding. They understand that there must be proper budget allocation for the provision of necessary resources. However, they reported that the overall internal revenue allocation (IRA) of the barangay is limited, thus limiting their DRRM fund.

In addition, the specific lack of ICT resources in the community was attributed by the participants to the unavailability of Internet and GSM connectivity provider in the area. To them, as connectivity is weak, it would make no sense to allocate money to ICT resources such as computer.

However, according to residents and local officials, they have previously requested private companies to improve connectivity in the area. The community’s request had been rejected because of limited market in the area. Hence, while enhancing connectivity can be an action point to resolve their vulnerability, the participants see such dilemma as beyond their control.

### Summary of results in study site B

Unlike in site A, both LLMC leader and members in site B are well performing. However, the participants, including the LLMC leader and members themselves, believe that this capacity can still be improved. Like in site A, they also think that providing compensation for the LLMC will enhance their performance. According to the participants, most of them cannot sacrifice their livelihood for participating in EWS-L activities such as data gathering. They emphasised that there is limited available livelihood in the area; hence, they must grab every opportunity (Table 3).

**TABLE 3:** Categorised capacities and vulnerabilities of site B.

Variable	Social relations	Physical resources	Trainings and learning resources	Appropriate skills	Governance and accountability
Capacities	Active LLMC leader and members	Available evacuation centre	Protocol trainings from PHIVOLCS	Surficial data gathering	Local government consistently provides development projects for the community
	-	Available communication tools (cell phone, handheld radio)	-	Dissemination skills	-
Vulnerabilities	Non-affected households do not care about the landslide hazard	Limited radio, GSM and Internet connectivity	Lack of training on sensor maintenance	Lack of computer skills	Tribal council is not involved in current EWS-L
	Low participation in community-led projects	Not all households are afforded electricity	No training yet on CBEWS-L management	Inconsistent surficial data gathering techniques	Poor DRRM budgeting in BLGU and MLGU levels
	-	-	-	Low basic literacy and numeracy	-

BLGU, Barangay local government unit; community-based early warning system for deep-seated landslides; DRRM, disaster risk reduction and management; EWS, early warning system; LLMC, local landslide monitoring committee; MLGU, municipal local government unit; PHIVOLCS, Philippine Institute of Volcanology and Seismology.

On the contrary, the participants believe that to improve their capacity in terms of LLMC participation, more members must be recruited. However, they believe that there is an underlying vulnerability: there are more residents who remain unaware of the EWS-L in their area than those who have previously been engaged by the project. One proof, they said, is the fact that their tribal council is actually not involved in the current system. This makes recruiting active members of the LLMC a challenge.

Compounding the problem is the location of houses across the barangay. Some residents, who are willing to participate in the EWS-L, live far from usual place of assemblies. Therefore, the participants said, their desire to keep their communities safe is hindered by the physical inaccessibility of interventions such as trainings.

In relation, the participants believe that to make trainings and learning resources effective, poor education in the community must be resolved. Specifically, as literacy and numeracy is a problem among many residents, it is difficult for them to make the most out of trainings and learning resources. Hence, improving basic level education is one action point that they identified. Alternatively, trainings and learning resources must be appropriated to their educational capacity.

In terms of physical resources, on the contrary, the participants believe that poor DRRM budgeting in the BLGU and MLGU levels can be resolved by actually increasing government funding for their area. They perceive that the lack of budget at the LGU level is a limiting factor to the official’s capacity to govern them. However, they do not know how their IRA could be increased.

Finally, like in site A, construction of cellular sites was identified as one action point that can be done to enhance the ICT resources of the community. However, they too had been rejected by private companies before; hence, they see this action point as highly impossible.

### Summary of results in study site C

For participants in site C, strengthening their indigenous practices can be a primary action point in enhancing their capacities and addressing their vulnerabilities. They believe that most of their capacities are rooted on their culture. If their culture would be upheld, their capacities can be further strengthened (Table 4).

**TABLE 4:** Categorised capacities and vulnerabilities of site C.

Variable	Social relations	Physical resources	Trainings and learning resources	Appropriate skills	Governance and accountability
Capacities	High participation in community activities such as DRRM trainings	Available evacuation centre	Protocol trainings from PHIVOLCS	Surficial data gathering	Indigenous DRRM governance practice
	Well-respected council of elders	Available computer at the barangay hall	Useful IECs on surficial data interpretation	High basic literacy and numeracy	-
	-	-	-	Indigenous dissemination skills	-
Vulnerabilities	Non-practice of imposed laws, especially among non-tribal members	Lack of communication tools	Lack of training on sensor maintenance	Lack of computer skills	Lack of documented DRRM policies because of oral tradition
	-	Limited radio, GSM and Internet connectivity	No training yet on CBEWS-L management	-	DRRM practice is centred on response, not so much on disaster preparedness
	-	Barangay hall is at-risk of landslide	-	-	-

CBEWS-L, community-based early warning system for deep-seated landslides; DRRM, disaster risk reduction and management; IEC, Information, education, and communication; PHIVOLCS, Philippine Institute of Volcanology and Seismology.

They also believe that although there are now residents who are not part of their indigenous tribe, there should be an effort to at least make them respect their traditions. This way, indigenous DRRM practices and rules can still be effective.

However, they maintain that documenting practices is not part of their culture. They said that they are able to preserve their DRRM practices even without documentation. One example is their practice of *pagsigaw* (shouting) of warnings from the top of slopes so that the message can be heard by everyone. They still implement this dissemination technique today.

Yet, the provincial LGU reiterated that although cultural practices are an advantage, it is also good to have written rules. Plans and policies are important in DRRM practice. They also said that to enhance planning and policy-making in the barangay, the provincial and municipal LGUs can provide trainings to the barangay LGU.

According to the participants, assistance to the local authorities in terms of planning and policy-making is both doable and advantageous. Firstly, site C is located near the municipality centre; hence, assistance can easily be given to local authorities. Second, they are convinced that plans and policies can enhance government funding. When there is proper funding, they believe that training and physical resources can easily be provided to them.

On the contrary, unlike in sites A and B, residents of site C are highly aware of the landslide hazard they are facing. Site C is situated in a mountainous region where shallow landslides frequently happen. Hence, encouraging people to participate in the EWS-L is, according to the participants, easy. They added that perhaps further provision of trainings would help improve this capacity.

However, although highly aware of the landslide risk, the participants expressed challenge with addressing the lack of computer skills among residents. They understand that the EWS-L uses ICTs and they also believe that ICTs make the system operation easier. However, they reported that acquiring computer skills can be a challenge especially because most site C residents are elderly. At their age, they are unwilling to adapt to new technologies. A solution, according to the participants, will be to engage younger residents.

Furthermore, they believe that one solution to the poor Internet and GSM connectivity in the area is to strengthen governance. They believe that engaging private entities will be easier if LGUs will lead the action.

### Summary of results in study site D1

Observable landslide indicators are present across the deep-seated landslide-prone area in site D1. Most residents are therefore aware of the presence of such hazard. This is what site D1 considers as one of the reasons why the LLMC is well performing. According to the participants, it is therefore a must that PHIVOLCS continues its efforts in educating the residents (Table 5).

**TABLE 5:** Categorised capacities and vulnerabilities of site D1.

Variable	Social relations	Physical resources	Trainings and learning resources	Appropriate skills	Governance and accountability
Capacities	Active LLMC leader and members	Available evacuation centre	Protocol trainings from PHIVOLCS	Surficial data gathering	Provincial government provides support to municipal and barangay LGUs in terms of DRRM
	-	Almost everyone has a cell phone for communication	-	High basic literacy and numeracy	-
Vulnerabilities	Differences or conflicts among families are apparent	No available office space for disaster monitoring	Lack of training on sensor maintenance	Only LLMC are skilled in surficial data gathering	DRRM policies at the barangay level are lacking
	-	Power outage is very common	-	Dissemination skills are limited to texting	-
	-	Limited radio, GSM and Internet connectivity	-	Lack of computer skills	-
	-	No available computer for the CBEWS-L	-	-	-

CBEWS-L, community-based early warning system for deep-seated landslides; DRRM, disaster risk reduction and management; LLMC, local landslide monitoring committee; LGU, local government units; PHIVOLCS, Philippine Institute of Volcanology and Seismology.

On the contrary, conflicts among residents are easily resolved. The participants said that they are all God-loving. Hence, it is easy to make peace within the community.

However, unlike enhancing social capacities and resolving social vulnerabilities, site D1 feels that addressing their insufficient physical resources is a huge challenge. For one, they cited the issue of low IRA, which they claimed not within their control. However, they think that educating government officials might help with funding.

From another perspective, local authorities of site D1 believe that enhancing DRRM funding in the barangay can be done through trainings. Specifically, the provincial and municipal governments are planning to conduct training on DRRM policy-making for both sites D1 and D2 because they belong to the same municipality. According to the PLGU and MLGU, trainings on policy-making will enable the BLGU to allocate its DRRM fund appropriately.

In addition, barangay DRRM policies are also foreseen to be helpful in enhancing the dissemination skills of the community. For instance, SMS is currently their means of communication but GSM signal is weak in the site. They believe that the use of siren or flags will be helpful. To do so, they said they need a resolution that standardises the use of these channels – how long is the alarm for each deep-seated landslide alert level, which flag signals which alarm?

### Summary of results in study site D2

Participation in the EWS-L and in decision-making is apparent in site D2 through active LLMC and open general assemblies. However, the participants feel that these capacities can still be enhanced. Particularly, they believe that the conflict between their livelihood and volunteer work needs to be resolved (Table 6).

**TABLE 6:** Categorised capacities and vulnerabilities of site D2.

Variable	Social relations	Physical resources	Trainings and learning resources	Appropriate skills	Governance and accountability
Capacities	Active LLMC leader and members	Available evacuation centre	Protocol trainings from PHIVOLCS	Surficial data gathering	Dissemination protocol has been institutionalised
	Residents are involved in decision-making during general assemblies	Multi-purpose hall serves as LLMC office	-	Surficial data interpretation	BDRRMC is functioning
	God-centred locals	No available computers	-	High basic literacy and numeracy	-
Vulnerabilities	None identified	Multi-purpose hall is within risk area	Lack of training on sensor maintenance	Lack of computer skills	DRRM policies at the barangay level are lacking
	-	Power outage is very common	-	Only LLMC are knowledgeable on surficial data gathering and interpretation	-
	-	Limited radio, GSM and Internet connectivity	-	-	-

BDRRMC, barangay disaster risk reduction and management committee; DRRM, disaster risk reduction and management; LLMC, local landslide monitoring committee; PHIVOLCS, Philippine Institute of Volcanology and Seismology.

According to the participants, the need to attend to their livelihoods hinders scheduling trainings and encouraging attendance which they believe would facilitate the CBEWS-L realisation. Specifically for site D2, harvest season comes every April and September. Hence, EWS-L processes, including capacity-building activities, are disrupted during these periods. A solution, according to the participants, is to take turns in performing surficial data gathering and to request PHIVOLCS to schedule the trainings accordingly. This would entail clarifying roles within the organisation and strengthening coordination with PHIVOLCS.

In relation, they also feel that although the LLMC is active, engaging other residents can be helpful to them. Specifically, like in D1, active participation of LLMC is attributed to their awareness of the landslide hazard. Hence, according to the participants, if more residents are made aware, more support for the LLMC members can be gained.

On the contrary, one social characteristic of the community which the participants think will help resolve their vulnerabilities is their being indigenous peoples (IP). They believe that filling the gaps in their physical resources can be addressed by capitalising on their indigenous identity. According to them, they can easily request for assistance from the government because IPs are given priority in their region.

Alternatively, they see the need to strengthen DRRM policies at the barangay level to help them gain financial support from the provincial and municipal government. However, participants from the barangay LGU themselves expressed insufficient of skills in policy-making, hindering the realisation of proper budgeting and later provision of necessary physical resources.

But this is not to say that the BLGU has not done DRRM-related policies at all. In fact, the participants emphasised that they now have a resolution standardising the use of *batingaw* or makeshift bells in disseminating deep-seated landslide alerts. Hence, with such expression of capacity, the proposed training on DRRM policy-making by the provincial and municipal governments for site D2 is seen not as a first step but as another step forward towards capacity improvement.

## Discussion and recommendations

### Heterogeneity and similarities of results

The site-specificity of capacities and vulnerabilities as posited by Davis et al. (2004) is apparent in the research results. Variations in the set of capacities and vulnerabilities for each site led to differences in identified action points.

For example, resolving challenge with literacy and numeracy has only been brought up in site B. This is primarily because it is only in site B where marginalisation is rampant, as described by the participants themselves. On the contrary, upholding cultural practices is an action point that has been uniquely identified in site C. Although three study sites (site B, C and D2) were selected for having indigenous residents, it is only in site C where traditional practices had been maintained. Evidently, differences in site conditions affected variations in capacities, vulnerabilities and corresponding action points.

However, although the overall set of capacities and vulnerabilities in one site may differ from the other, certain existing capacities or vulnerabilities were observed in more than one site. For example, all sites mentioned the following capacities:

- availability of evacuation centres
- EWS trainings
- skills in surficial data gathering.

The last two items are apparent because these are the main themes discussed during past project capacity-building activities. Other capacities and vulnerabilities existing in more than one study site are as follows:

- active LLMC leaders or members
- lack of ICT materials
- poor ICT infrastructure
- lack of ICT skills
- varying challenges in DRRM practice at the barangay level.

Apparently, organisational, material and governmental challenges are common in all study sites. It is evident from these results that although the study sites are unique, certain characteristics can be similar. Hence, although it is true that there is no single realising framework for establishing an EWS (Macherera & Chimbari 2016; Zia & Wagner 2015), there can be action points which are applicable to one site that can also be applicable to another.

For example, the provision of incentives for the LLMC was identified as facilitating factor to enhancing social relations in sites A, B and D2. The provision of trainings on DRRM planning and policy-making for local officials in the barangay was also mentioned in sites C, D1 and D2. Resolving problems with connectivity by lobbying with private companies was another common action point for sites A, B and C.

It is therefore recommended that all possible action points should be exhausted, from where appropriate ones can be selected for each site. However, it is in the careful selection of action points where the difference lies. Although action points are similar at the surface, underlying situations may affect the overall strategy in one site.

### Existing indirect factors

Enabling the capacities and compounding the vulnerabilities identified as direct factors to the CBEWS-L implementation are underlying capacities and vulnerabilities in the study sites. These indirect factors are as follows:

- Local economy (livelihood and income levels) – influences social relations because the need to attend to livelihood hinders LLMC and community participation in the EWS-L, such as in the case of sites A, B and D2.
- Education (basic literacy and numeracy) – a challenge in providing trainings and learning resources in site B.
- Culture (indigenous knowledge and practices) – how DRRM practices are established in site C, hence hindering the institutionalisation of the CBEWS-L.
- Awareness (risk and project awareness) – hinders the credibility and participation of LLMC in site A, and facilitates local participation in sites C, D1 and D2.
- Physical environment (location of residence and access to facilities) – like local economy, this influences LLMC and community participation specifically in site B.
- Government funding (IRA or DRRM fund) – dictates provision of physical resources and training in site A, affects governance and accountability in Site B and determines provision of physical resources in site D1.
- Local investment (private investment to infrastructure) – lack of network infrastructure hinders availability of ICT materials in sites A, B and C.

Conditions falling under these categories do not directly facilitate or hinder the CBEWS-L realisation; rather, they influence the facilitating or hindering factors per se. This finding agrees well with the progression of vulnerability into dynamic pressured and root causes, posited by Wisner et al. (2003).

Therefore, it is recommended that while action points can be shared among sites, strategies should be site-specific. For instance, incentivising the LLMC in sites A, B and D2 to enhance LLMC and community participation may yield varying results. An incentive system for site A, where risk awareness is a problem, might still not encourage residents to become LLMC members. Similarly, incentives for site B may be more effective because they are more aware of their risk; however, the issue of education will still hinder the capacity-building needed for LLMC members. On the contrary, incentives might serve as a cherry on top to the already well-performing LLMC of site D2.

In other words, the combination of action points, their order and manner of implementation is highly dependent on present and pressing community conditions, especially because some of the said community conditions are spatially distant or are not within the control of the community, the local authorities or PHIVOLCS.

To consider all possible factors, it is recommended that a more systematic use of the PAR model is done. This means that communities must first be well oriented with the notion of progression of capacities and vulnerabilities. Then, they should be able to identify clear linkages among factors that may facilitate or hinder the CBEWS-L realisation. In doing so, even the root causes, which are deemed systemic, can be made known.

### Social and political aspects of capacities and vulnerabilities

Contrast among the sites’ capacities and vulnerabilities is most apparent in terms of social relations and governance and accountability. This indicates that establishing a CBEWS-L must not only be technological, but should also be both social and political.

Previous literature states that the effectiveness of an EWS (or any DRRM effort for that matter [Allen 2006]) cannot merely rely on its technical component, but social factors must be addressed as well (Fathani & Karnawati 2012; Karnawati et al. 2011; Fischer et al. 2012). The UNISDR checklist for the establishment of EWS (UNISDR 2005), on the contrary, lists ‘Effective Governance and Institutional Arrangements’ as a cross-cutting issue that should be addressed to ensure sustainability. Similarly, government units are encouraged to get involved in DRRM researches so that the results can directly influence policies (Mercer et al. 2008).

Therefore, aside from uncovering social and political capacities and vulnerabilities, a socio-technical approach in strategy-building must be taken as well. In EWS, a socio-technical approach entails the use of technology which is appropriate to psychosocial site conditions (Karnawati et al. 2011).

In the case of this study, the possibility of using technological solutions in the CBEWS-L was given emphasis. However, it has been clear in the results that there are several gaps in community resources in terms of ICTs. These gaps are not just limited to material resources, but to the willingness, learning resources and skills of communities, compounded by political conditions as well.

Following the ICT4D perspective (Beardon 2008), it is evident that the social and political gaps in the study sites must be considered in the development of technologies which communities can use themselves. Resolving social and political gaps can be a long process. Furthermore, changing social conditions, particularly organisational settings (Adman and Warren 2000), may result to changing technological needs.

Hence, it is recommended that the development of technologies for the CBEWS-L should be made modular. A modular approach and phased process allows for emergence of new requirements (Waller et al. 2006). In this way, a more sustainable CBEWS-L implementation can be achieved.

### Multiplicity of stakeholders

The complexity of capacities and vulnerabilities in the study site necessitates the participation of multiple stakeholders in implementing the CBEWS-L. For instance, funding and provision of other services and resources are deemed by the communities as dependent mostly on the government. Moreover, local investment, which is highly dependent on private sectors, was also seen as a factor for CBEWS-L success, especially in terms of communication utilities.

These findings highlight the need for linkages and collaborations where resources are insufficient, as suggested in literature (Parkash 2012). As seen from the results, disasters may not be the only priority of a community. Hence, tackling the issue of DRRM given the set of other community needs may require more resources which a single institution may not be able to fully support. One form of such linkage is cross-programme integration such that continuous support for DRRM can be achieved.

Alternatively, previous literature indicates that the use of simpler technologies where resources are limited can be successful (Abon et al. 2012; Fathani & Karnawati 2012; Manalo 2013; Fischer et al. 2012). Yet, this does not mean that local investment can be left unresolved, especially if, as implied by Parkash (2012), resolving resource problems through linkages may lead to sustainability.

Therefore, a multi-stakeholder approach to the CBEWS-L implementation is necessary. Furthermore, the multiplicity of stakeholders to be engaged must be ensured throughout the CBEWS-L realisation process – from the design of the system, its development and its actual implementation.

### An emphasis on risk awareness

In introducing this study, we argued that establishing a CBEWS-L necessarily includes the four PEWS elements (UNISDR 2005). We also argued that, among the four elements, establishing risk knowledge is a prerequisite to further developments. Hence, we conducted capacity and vulnerability assessment.

The results of such assessment now further prove the importance of risk knowledge. The lack of risk and EWS-L awareness among locals was deemed by the research participants as a hindrance to the CBEWS-L vision. Awareness was found to be an underlying factor to social relations. Particularly, lack of awareness among locals results in low participation in the EWS-L.

As mentioned, deep-seated landslides are characterised by very slow movements (Kaunda 2010). Hence, deep-seated landslides are often left unnoticed. This could also explain the low risk awareness reported in three study sites.

The nature of deep-seated landslides therefore further highlights the necessity of tackling risk knowledge prior to the CBEWS-L establishment. To date, no comprehensive risk knowledge generation has been done in any of the EWS-L sites; hence, a community risk assessment is highly recommended. Moreover, to uncover root causes, the PAR model should be used as a conceptual framework for future studies.

### Limitations of the study

The study was qualitative and participatory, and the unit of analysis is the community. Hence, the expected results are site-specific and not generalisable. While this is the case, five case studies were purposively sampled to cover different situations of communities in the Philippines. Results showed similarities and differences across all five cases. Quantitative data were not gathered; hence, the study can be validated or falsified by more experimental designs.

Moreover, the research results only described the communities’ capacities and vulnerabilities, but did not indicate actions that the communities must take. In addition, while the research adhered to the PEWS framework (UNISDR 2005), the main focus was on the operational side of the system, namely (1) monitoring, warning and service and (2) dissemination and communication because these were the current ICT development priority of the project.

## Conclusion

Through the use of participatory assessments, capacities and vulnerabilities of five barangays in establishing EWSs for deep-seated landslides were identified in our study, along with action points to address them. Identified situations were highly site-specific. However, similarities in themes allow for strategic solutions in establishing CBEWS-L. Solutions were only identified, and in-depth planning of strategies is still recommended.

While the initial focus of the study is to understand the capacity of the community of operating EWS-L consisting of ICT, framing the CBEWS-L realisation as both a social and political endeavour is necessary. Not only should the technological capacities and vulnerabilities of the study site be examined, but underlying factors should be considered as well.

A more comprehensive community risk assessment that uses the PAR model is still needed. Furthermore, contin-uously using a participatory approach to the CBEWS-L implementation is necessary so as to include the multiple stakeholders who may be key to the success and sustainability of the system.
